# Mechanism underlying vascular remodeling in relation to circulating CD34-positive cells among older Japanese men

**DOI:** 10.1038/s41598-022-26089-y

**Published:** 2022-12-17

**Authors:** Yuji Shimizu

**Affiliations:** 1 Department of General Medicine, Nagasaki University Graduate School of Biomedical Sciences, Nagasaki-shi, Sakamoto 1-12-4, Nagasaki, 852-8523 Japan; 2 Department of Cardiovascular Disease Prevention, Osaka Center for Cancer and Cardiovascular Diseases Prevention, Osaka, Japan

**Keywords:** Diagnostic markers, Predictive markers, Angiogenesis, Cardiovascular diseases

## Abstract

Development of structural atherosclerosis, an established cardiovascular risk factor, requires hematopoietic stem cells known as CD34-positive cells. However, an inverse association between circulating CD34-positive cell count and cardiovascular disease has been reported. These studies evoke a contradiction: characteristics associated with a low risk of developing structural atherosclerosis are also associated with a high risk of cardiovascular disease. To clarify the mechanisms underlying vascular remodeling, we conducted several epidemiological studies of Japanese men aged 60 to 69 years who participated in annual health check-ups. The present study summarizes those epidemiological studies and adds some discussion. From the perspective of endothelial repair activity, there are significant differences between functional versus structural atherosclerosis. Aggressive endothelial repair increases both functional and structural atherosclerosis. Deficient endothelial repair related to a shortage of CD34-positive cells due to consumption furthers functional atherosclerosis but not structural atherosclerosis. Therefore, the lack of structural atherosclerosis does not always reflect a favorable condition for the endothelium. Although further investigation is necessary, the present study suggests that higher endothelial repair activity that leads to structural atherosclerosis might have a beneficial effect on vascular health among older men.

## Introduction

Upon vascular injury, platelets become activated and take on an important role in vascular homeostasis^1^. Activated platelets induce proliferation of CD34-positive cells^2^ and their differentiation into megakaryocytes, a known source of platelets^3^, as well as endothelial cells^4^, macrophages, and foam cells^2^. Since macrophages^5^ and foam cells^6^ contribute to the development of pathological atherosclerosis, CD34-positive cells might be necessary for the development of structural atherosclerosis.

However, circulating CD34-positive cell count is reported to be inversely associated with cardiovascular disease^7–9^ while increased carotid intima-media thickness (CIMT), which indicates developing structural arterial stiffness, is reported to be associated with cardiovascular disease^10,11^.

Therefore, these studies suggest a contradiction, namely that characteristics associated with a low risk of developing structural atherosclerosis are also associated with a high risk of cardiovascular disease. Although CD34-positive cells contribute to the development of structural atherosclerosis, they also contribute to the maintenance of the microcirculation by promoting angiogenesis^12^ and neovascularization^13^. Inhibition of angiogenesis induces hypertension^14^, which is the strongest cardiovascular risk factor^15^. Therefore, circulating CD34-positive cell count could be inversely associated with cardiovascular disease because it reflects active maintenance of the microcirculation. Furthermore, because CD34-positive cells can differentiate into endothelial cells^4^, a deficiency of CD34-positive cells could result in deficient endothelial repair, or functional atherosclerosis.

Thus, we hypothesize that there is a novel mechanism in which CD34-positive cells play an important role in vascular remodeling, such as endothelial repair and maintenance of the microcirculation. To clarify this novel mechanism, we have conducted several epidemiological studies.

## Materials and methods

To investigate the role of circulating CD34-positive cells in mechanisms of vascular remodeling, we have performed a circulating CD34-positive cell-related survey. This survey was conducted as an addition to the annual health check-up recommended by the Japanese government. The detail of this survey has been described elsewhere^16^.

### Study population

To measure circulating CD34-positive cell count, approximately 30 min is required for one sample. Measurement of circulating CD34-positive cell count requires a fresh sample, within 24 h of blood collection. Therefore, CD34-positive cell count can be measured in a maximum of 20 samples each day. Thus, we limited the measurement of CD34-positive cell count to men aged 60–69 years who participated in an annual health-up in the city of Goto and the town of Saza. Both are located in Nagasaki Prefecture, in western Japan. The city of Goto is located on a remote island. We evaluated handgrip strength and cardio-ankle vascular index (CAVI) only in the city of Goto. The town of Saza is known as a bedroom community adjacent to the city of Sasebo. CIMT was evaluated in both study locations.

Those studies were approved by the ethics committee of the Nagasaki University Graduate School of Biomedical Sciences (project registration numbers, 14051404-1 to 14051404-13). Written consent forms were made available to ensure that the participants understood the objective of the study. Informed consent was obtained from all participants. All procedures performed in this study were in accordance with the ethical standards of the institutional research committee and the 1964 Declaration of Helsinki and its later amendments.

### Data collection

Because the target population of our circulating CD34-positive cell–related survey was limited to men within a narrow age range, our study is the largest general population-based study in the world that deals with circulating CD34-positive cell count among general men within narrow range of age.

#### Measurement of circulating CD34-positive cells

Fresh samples from heparin sodium tubes were used to measure CD34-positive cell count within 24 h of blood collection. Automated software on the Becton Dickinson Biosciences (BD) FACSCanto™ II system was used in accordance with the International Society for Hematotherapy and Graft Engineering (ISHAGE) guidelines^17^.

#### Structural arterial stiffness assessment

An experienced vascular examiner measured CIMT in the left and right common carotid arteries using a LOGIQ Book XP with a 10-MHz transducer (GE Healthcare, Milwaukee, WI, USA). Mean and maximum left and right common CIMT values were calculated using automated digital edge-detection software (Intimascope; MediaCross, Tokyo, Japan) and a previously described protocol^18^. Intimascope software was used to increase the accuracy and reproducibility of CIMT measurements. This software semi-automatically recognizes the edges of the internal and external membranes of the artery and automatically determines distances at a sub-pixel level, estimated to be 0.01 mm^19^.

#### Functional arterial stiffness assessment

Brachial-ankle pulse wave velocity (PWV) measurements are generally used to evaluate functional arterial stiffness. Since PWV measurements can be strongly affected by blood pressure^20^, cardio-ankle vascular index (CAVI) was recently developed in Japan to avoid the confounding effects of blood pressure^21^. In the current study, CAVI was determined using a VaSera VS-1000 vascular screening system (Fukuda Denshi, Tokyo, Japan) with the participant resting in a supine position.

#### Handgrip strength assessment

Handgrip strength was determined using a handgrip dynamometer (Smedley, Matsumiya Ika Seiki Seisakujo, Tokyo, Japan) as grip strength from two measurements obtained for each hand. The maximum value from each hand was used.

#### Definition of active arterial wall thickening, structural atherosclerosis, and hypertension

Given that the present study used Intimascope, we defined active arterial wall thickening as an increase in CIMT of ≥ 0.01 mm/year. We defined structural atherosclerosis as CIMT ≥ 1.1 mm. Hypertension was defined as systolic blood pressure ≥ 140 mmHg, diastolic blood pressure ≥ 90 mmHg, or both.

## Results

We have performed several studies about circulating CD34-positive cells and vascular remodeling based on data from the city of Goto and the town of Saza. Parts of those studies are described below.

### Platelets, circulating CD34-positive cells, and hypertension^22^ (Fig. 1)

**Figure 1:**
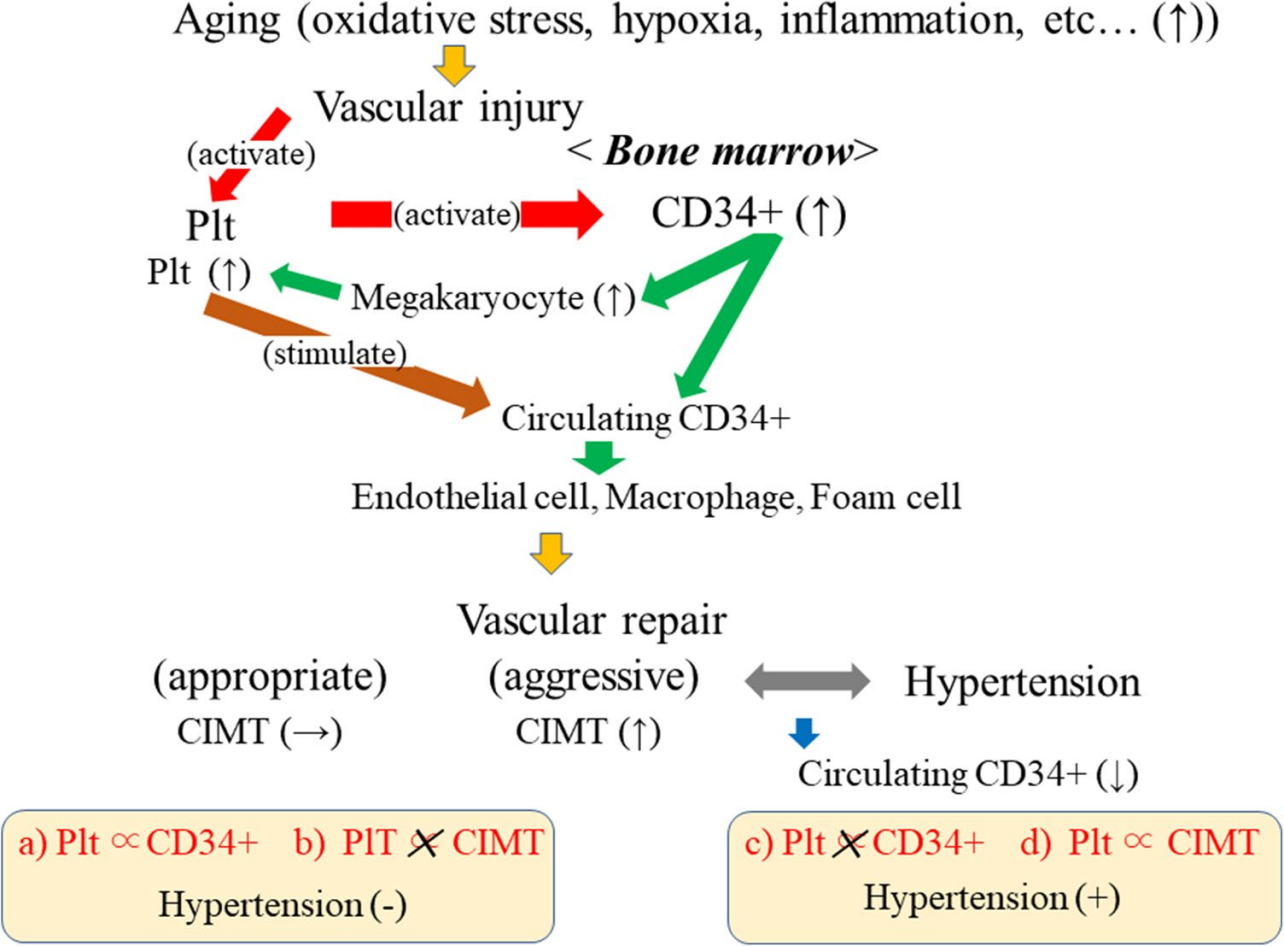
Platelet, circulating CD34-positive cell, and hypertension^22^. Associations shown in red (a–d) were observed in previous study. Associations (a,b) were observed among non-hypertensive men and (c,d) were observed among hypertensive men. *CD34*+ CD34-positive cell, *CIMT* carotid intima-media thickness, *Plt* platelet.

To clarify the role of platelets and circulating CD34-positive cells in hypertension and structural atherosclerosis, a cross-sectional study with 567 Japanese men aged 60–69 years was conducted^22^. Independent of known cardiovascular risk factors, platelet count was found to be significantly positively correlated with circulating CD34-positive cell count (standardized parameter estimate (β) = 0.26, p < 0.001) (Fig. 1a) but not with CIMT (β =  − 0.05, p = 0.356) (Fig. 1b) in men without hypertension. In men with hypertension, no significant correlation was observed between platelet count and circulating CD34-positive cell count (β = 0.11, p = 0.119) (Fig. 1c), but a significant positive correlation was observed between platelet count and CIMT (β = 0.19, p = 0.008) (Fig. 1d).

### Circulating CD34-positive cells and active arterial wall thickening^23^ (Fig. 2)

To clarify the influence of circulating CD34-positive cells on yearly progression of CIMT, we conducted a prospective study of 363 men aged 60–69 years who participated in our survey at least twice during the observation period^23^. The follow-up period was 2.20 ± 0.53 years.

Independent of known confounding factors, baseline hypertension is significantly positively associated with baseline atherosclerosis but not with active arterial wall thickening. The adjusted odds ratio (OR) and 95% confidence interval (CI) of baseline atherosclerosis for baseline hypertension was 2.41 (1.28, 4.78). The adjusted OR and 95% CI of active arterial wall thickening for baseline hypertension was 1.22 (0.78, 1.92). Baseline atherosclerosis was significantly inversely associated with active arterial wall thickening (adjusted OR 0.24; 95% CI (0.11, 0.52)).

**Figure 2:**
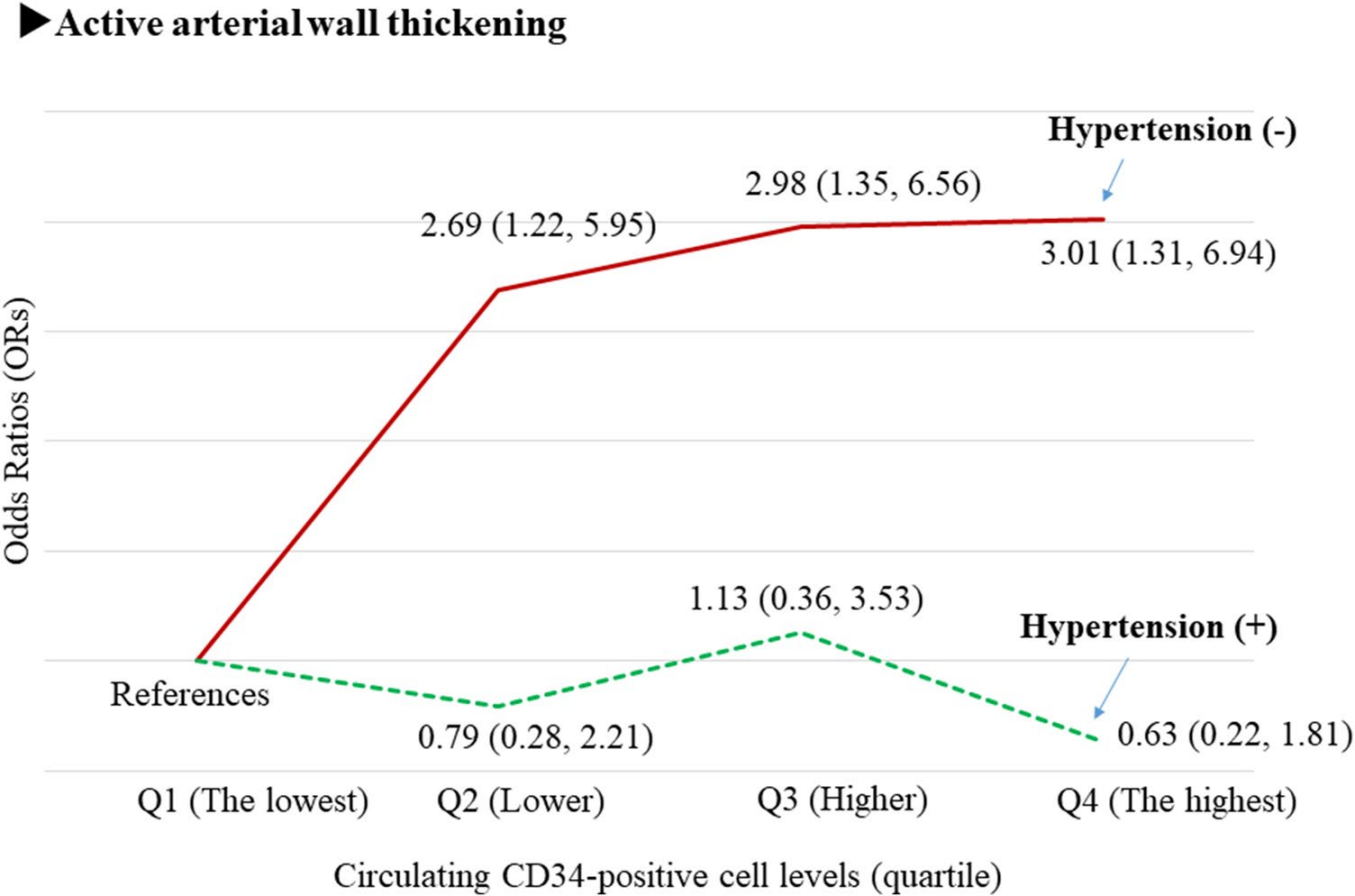
Associations between circulating CD34-positive cell levels and active arterial wall thickening^23^. Active arterial wall thickening: yearly increased CIMT value at and above 0.01 mm. Q1: The lowest quartile level. Q2: Lower quartile level, Q3: Higher quartile level. Q4: The highest quartile level.

Furthermore, circulating CD34-positive cell count was significantly positively associated with active arterial wall thickening only in men without hypertension. With the lowest quartile of circulating CD34-positive cell count (Q1) as the referent group, the fully adjusted OR and 95% CI of active arterial wall thickening was 2.69 (1.22, 5.95) for the second lowest quartile (Q2), 2.98 (1.35, 6.56) for the second highest quartile (Q3), and 3.01 (1.31, 6.94) for the highest quartile (Q4) among men without hypertension. The corresponding values for men with hypertension were 0.79 (0.28, 2.21), 1.13 (0.36, 3.53), and 0.63 (0.22, 1.81), respectively (Fig. 2).

### Structural arterial stiffness, functional arterial stiffness, and circulating CD34-positive cells^24^ (Fig. 3)

Circulating CD34-positive cells could differentiate into endothelial cells^4^, macrophages, and foam cells^2^. Those cells are essential for the development of structural atherosclerosis, but not functional atherosclerosis. Therefore, circulating CD34-positive cell count could influence the development of both structural and functional atherosclerosis. We performed a cross-sectional study of 249 Japanese aged 60–69 years with available data on structural arterial stiffness (CIMT) and functional arterial stiffness (CAVI)^24^.

**Figure 3:**
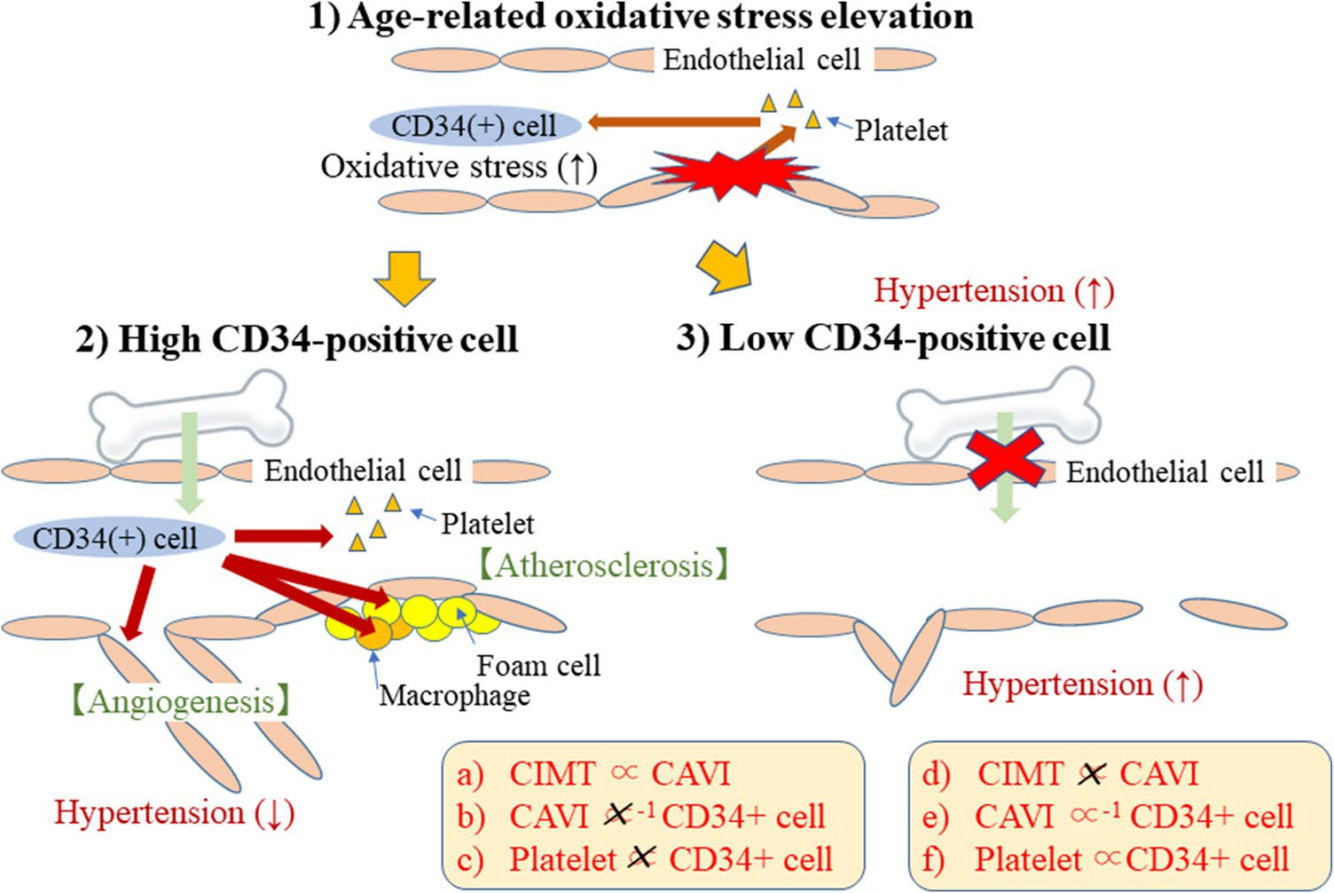
Potential vascular mechanism that underlying the adaptation for age-related oxidative stress^24^. Associations shown in red (a–d) were observed in previous study. *CAVI* cardio ankle vascular index, *CIMT* carotid intima-media thickness.

Independent of known confounding factors, for participants with high circulating CD34-positive cell count (at or above the median), CIMT was correlated with CAVI (β = 0.22, p = 0.028) (Fig. 3a), but not in participants with low circulating CD34-positive cell count (below the median) (β =  − 0.02, p = 0.865) (Fig. 3d).

Furthermore, no significant correlation between CAVI and circulating CD34-positive cell count was observed among participants with high circulating CD34-positive cell count (β =  − 0.04, p = 0.638) (Fig. 3b). However, a significant inverse association was observed between CAVI and circulating CD34-positive cell count among participants with low circulating CD34-positive cell count (β =  − 0.22, p = 0.014) (Fig. 3e).

In addition, we also evaluated the correlation between platelet and circulating CD34-positive cell counts because such a correlation could reflect endothelial repair activity^22^. While no significant correlation was observed between platelet and circulating CD34-positive cell counts among participants with high circulating CD34-positive cell count (simple correlation coefficient (r) =  − 0.02 and p = 0.848) (Fig. 3c), a significant positive correlation was observed for those with low circulating CD34-positive cell count (r = 0.23, p = 0.009) (Fig. 3f).

### Associations between structural arterial stiffness and chronic kidney disease (CKD) by circulating CD34-positive cell count^25^ (Fig. 4)

CKD is reported to be associated with structural atherosclerosis^26^ and circulating CD34-positive cells play an important role in the progression of structural atherosclerosis^23,24^. Thus, circulating CD34-positive cell count could influence the association between structural arterial stiffness and CKD.

**Figure 4:**
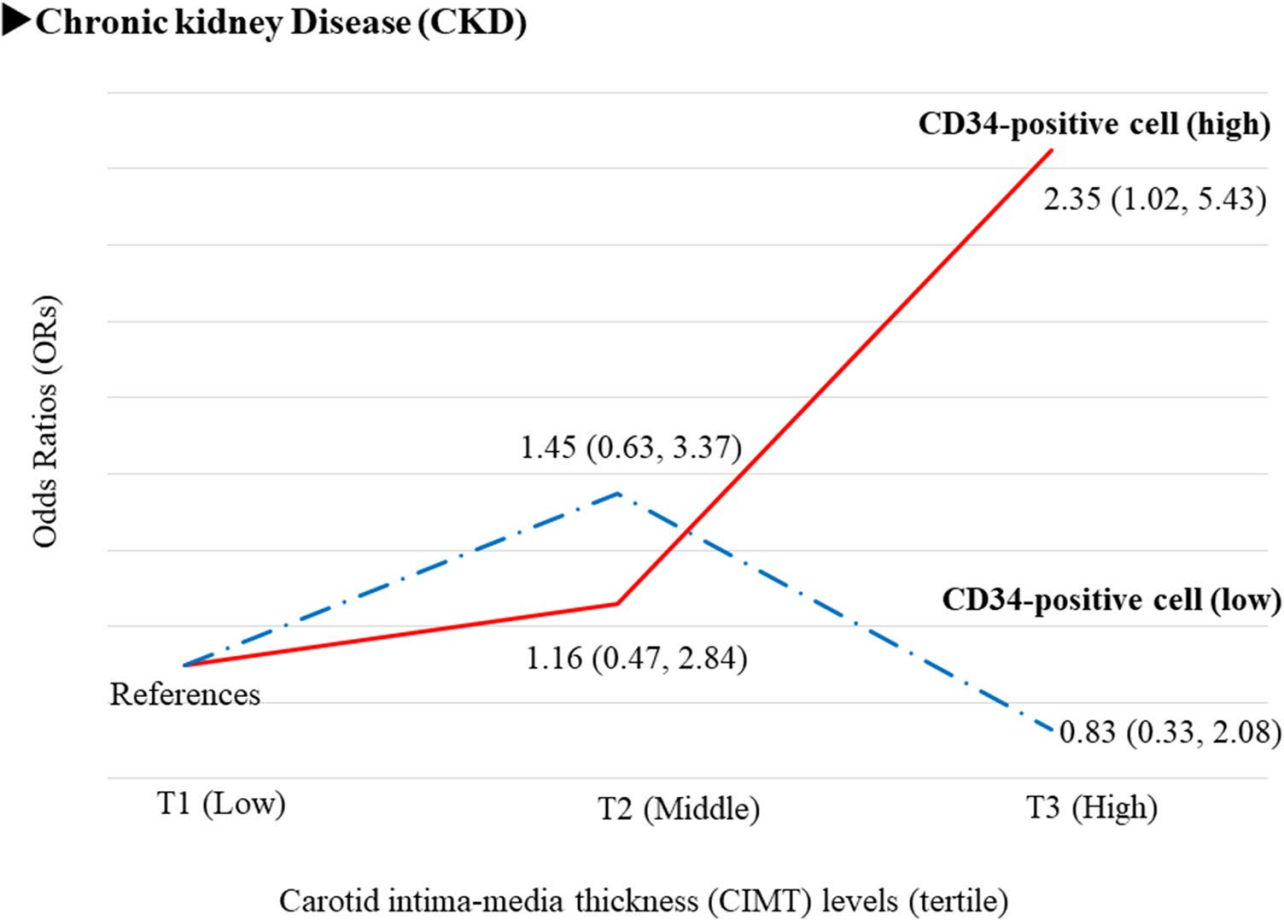
Association between carotid intima-media thickness (CIMT) and chronic kidney disease (CKD)^25^. T1:The lowest tertile level. T2: Middle tertile level, T3: The highest tertile level.

We conducted a cross-sectional study of 570 Japanese men aged 60–69 years^25^. Independent of known cardiovascular risk factors, CIMT was significantly positively associated with CKD only among participants with high circulating CD34-positive cell count (at or above the median). With participants in the lowest tertile (T1) of CIMT as the referent group, the fully adjusted OR and 95% CI of CKD for high CD34-positive cell count was 1.16 (0.47, 2.84) for the middle tertile (T2) and 2.35 (1.02, 5.43) for the highest tertile (T3). The corresponding values for low CD34-positive cell count were 1.45 (0.63, 3.37) and 0.83 (0.33, 2.08), respectively^25^ (Fig. 4).

### Associations among gamma-glutamyl transpeptidase (γ-GTP), hypertension, and structural atherosclerosis^27^ (Fig. 5)

Oxidative stress causes hypertension^28^ and atherosclerosis^29^. Serum gamma-glutamyl transpeptidase (γ-GTP) could act as a marker of oxidative stress^30,31^. To evaluate the influence of circulating CD34-positive cell count on the association between oxidative stress and hypertension and between oxidative stress and atherosclerosis, a cross-sectional study with 562 men aged 60–69 years was conducted^27^.

**Figure 5:**
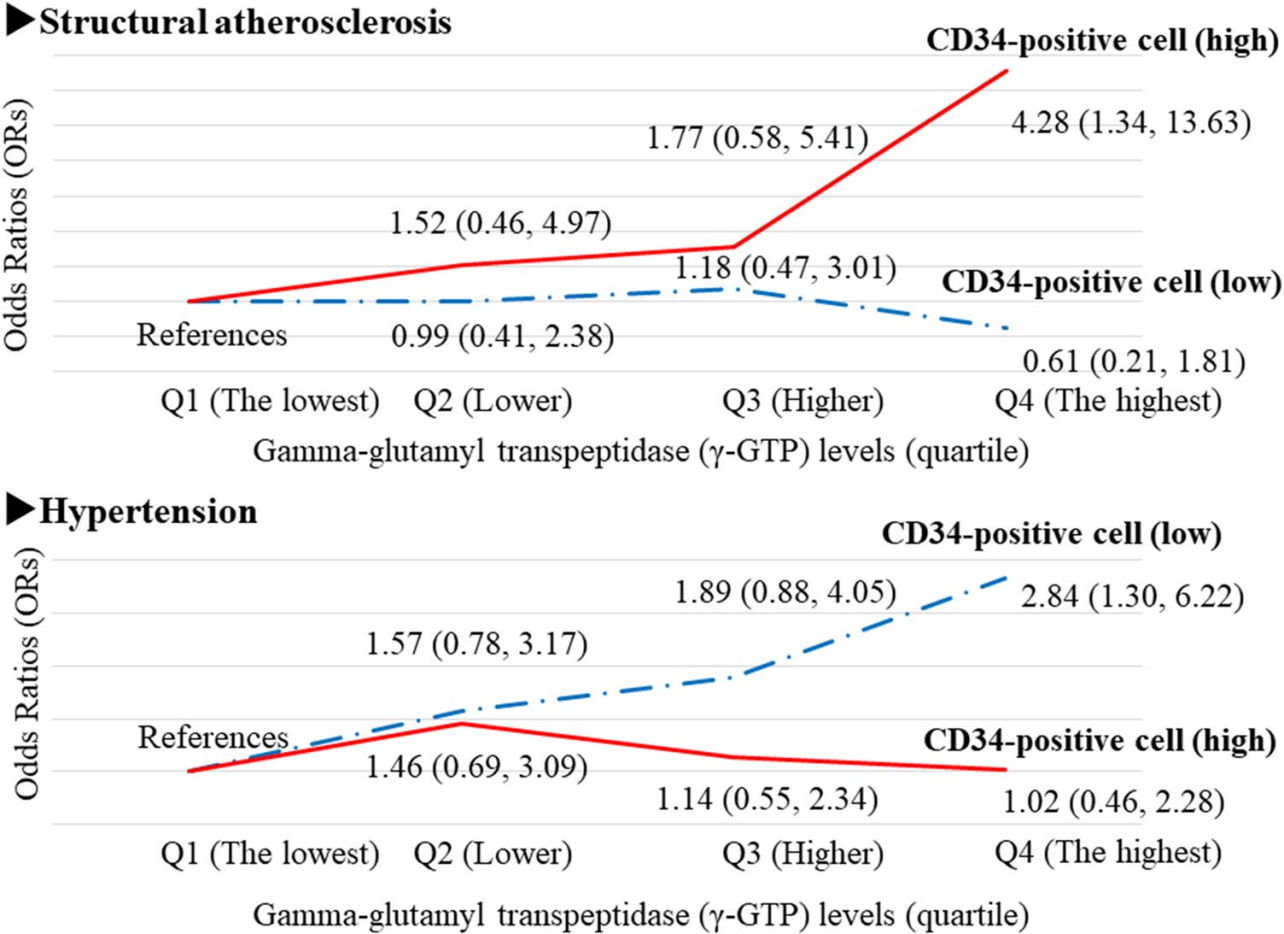
Association among gamma-glutamyl transpeptidase (γ-GTP), hypertension, structural atherosclerosis^27^. Q1: The lowest quartile level. Q2: Lower quartile level, Q3: Higher quartile level. Q4: The highest quartile level.

Independent of known confounding factors, hypertension was significantly positively associated with structural atherosclerosis; the adjusted OR (95% CI) was 2.09 (1.30, 3.35). The effect of circulating CD34-positive cell count (low or high) on the association between γ-GTP and structural atherosclerosis was evaluated. In participants with high circulating CD34-positive cell count (at or above the median), γ-GTP was significantly positively associated with structural atherosclerosis, but this association was not observed in participants with low circulating CD34-positive cell count (below the median). With participants in the lowest γ-GTP quartile (Q1) as the referent group, the fully adjusted OR and 95% CI of structural atherosclerosis for high CD34-positive cell count was 1.52 (0.46, 4.97) for Q2, 1.77 (0.58, 5.41) for Q3, and 4.28 (1.34, 13.63) for Q4. The corresponding values for low CD34-positive cell count were 0.99 (0.41, 2.38), 1.18 (0.47, 3.01), and 0.61 (0.21, 1.81), respectively.

In addition, we also evaluated the effect of circulating CD34-positive cell count (low or high) on the association between γ-GTP and hypertension. No significant association was observed between γ-GTP and hypertension in participants with high circulating CD34-positive cell count (at or above the median). A significant positive association was observed for those with low circulating CD34-positive cell count (below the median). With participants in the lowest γ-GTP quartile (Q1) as the referent group, the fully adjusted OR and 95% CI of hypertension for high CD34-positive cell count was 1.46 (0.69, 3.09) for Q2, 1.14 (0.55, 2.34) for Q3, and 1.02 (0.46, 2.28) for Q4. The corresponding values for low CD34-positive cell count were 1.57 (0.78, 3.17), 1.89 (0.88, 4.05), and 2.84 (1.30, 6.22), respectively.

### Handgrip strength and hypertension in relation to circulating CD34-positive cell count^32^ (Fig. 6)

A positive association between handgrip strength and hypertension has been reported^33,34^. Since hypertension has a common etiology with structural atherosclerosis^28,29^, circulating CD34-positive cell count could influence the association between handgrip strength and hypertension.

**Figure 6:**
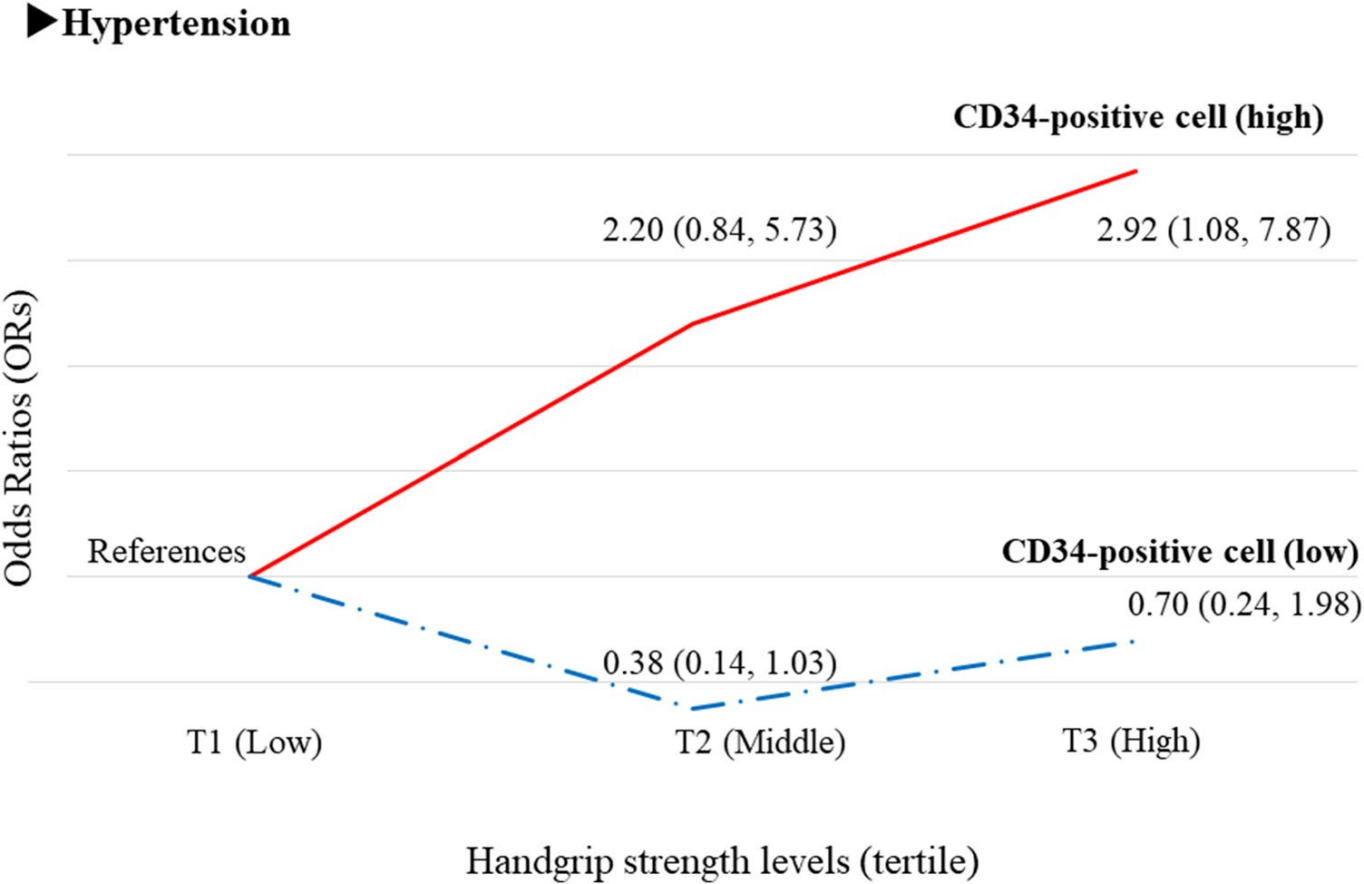
Associations between handgrip strength levels and hypertension^32^. T1: The lowest tertile level. T2: Middle tertile level, T3: The highest tertile level.

We conducted a cross-sectional study of 257 Japanese men aged 60–69 years with available data on handgrip strength^32^. Independent of known cardiovascular risk factors, handgrip strength was significantly positively associated with hypertension only among participants with high circulating CD34-positive cell count (at or above the median). With participants in the lowest tertile of handgrip strength (T1) as the referent group, the fully adjusted OR and 95% CI of hypertension for high CD34-positive cell count was 2.20 (0.84, 5.73) for T2 and 2.92 (1.08, 7.87) for T3. The corresponding values for low CD34-positive cell count were 0.38 (0.14, 1.03) and 0.70 (0.24, 1.98), respectively.

## Discussion

The main findings of present study are that aggressive endothelial repair increases both functional and structural atherosclerosis, while deficient endothelial repair furthers functional atherosclerosis but not structural atherosclerosis.

### Platelet, circulating CD34-positive cells, and hypertension

In conjunction with platelets, CD34-positive cells play an important role in vascular repair^1–6^. Vascular injury stimulates proliferation of both platelets and CD34-positive cells. Thus, platelet count could be positively correlated with circulating CD34-positive cell count.

However, with aggressive vascular repair, many CD34-positive cells differentiate into mature, CD34-negative cells such as megakaryocytes^3^, endothelial cells^4^, macrophages, and foam cells^2^. Aggressive vascular repair causes a reduction in circulating CD34-positive cell count due to consumption. However, platelet count is much higher than CD34-positive cell count in peripheral blood. Megakaryocytes are a source of platelets. Reductions in circulating CD34-positive cell count due to consumption might not have a strong effect on platelet count. Therefore, with aggressive vascular repair, the positive association observed between platelet and CD34-positive cell counts could disappear. In addition, the influence of consumption might be smaller for platelets than for circulating CD34-positive cells during aggressive endothelial repair. Furthermore, activated vascular repair induces platelet proliferation by stimulating circulating CD34-positive cells to differentiate into megakaryocytes^3^. Since megakaryocytes are a source of platelets and aggressive endothelial repair increases structural arterial stiffness, platelet count could be significantly positively correlated with CIMT, a marker of structural atherosclerosis.

To test this hypothesis, we performed a cross-sectional study of 567 Japanese men aged 60–69 years. Since hypertension injures the vasculature and induces aggressive endothelial repair, analyses were stratified by hypertension status. Potential mechanisms that explain the associations observed in this study are shown in Fig. 1. Among men with hypertension, platelet count was revealed to be significantly positively associated with CIMT (Fig. 1d), but not with circulating CD34-positive cell count (Fig. 1c). Among men without hypertension, no significant association between platelet count and CIMT was observed (Fig. 1b), but platelet count was positively associated with circulating CD34-positive cell count (Fig. 1a)^22^. This study shows that platelet count acts as an indicator of vascular repair and hypertension reduces circulating CD34-positive cell count due to consumption.

### Circulating CD34-positive cells and active arterial wall thickness

Although active arterial wall thickening (development of structural arterial stiffness) requires CD34-positive cells, no studies have reported a direct association between circulating CD34-positive cell count and active arterial wall thickening. To evaluate the influence of circulating CD34-positive cells on yearly progression of arterial stiffness (active arterial wall thickening), we defined active arterial wall thickening as an annual increase in CIMT ≥ 0.01 mm in our analysis stratified by hypertension status^23^. In this study, we found a significant positive association between active arterial wall thickening and circulating CD34-positive cell count only among participants without hypertension (Fig. 2). Hypertension, which induces aggressive vascular repair, acts as a confounding factor on the association between active arterial wall thickening and circulating CD34-positive cell count.

### Active arterial wall thickening and baseline structural atherosclerosis

Because hypertension stimulates aggressive vascular repair, hypertension is known to be positively associated with structural atherosclerosis^27,35^. Aggressive vascular repair reduces circulating CD34-positive cell count due to consumption. Since circulating CD34-positive cells are required for active arterial wall thickening, participants with baseline atherosclerosis might have a lower chance of getting active arterial wall thickening. We found a significant inverse association between active arterial wall thickening and baseline structural atherosclerosis^23^. Therefore, lack of yearly CIMT progression is not always a favorable condition for vascular health. A previous study with 36,984 participants, which reported no significant association between yearly CIMT progression and cardiovascular events^36^, supports our results.

### Baseline hypertension, baseline structural atherosclerosis, and active arterial wall thickness

Hypertension induces aggressive vascular repair that reduces circulating CD34-positive cell count. Because a shortage of circulating CD34-positive cells lowers the chance of active arterial wall thickening, no significant association between baseline hypertension and active arterial wall thickening was observed in our prior study, despite a significant positive association between baseline structural atherosclerosis and baseline hypertension^23^. Evaluation of vascular condition only based on structural atherosclerosis is not sufficient. Since deficient vascular repair does not influence structural arterial stiffness, another parameter that indicates deficient vascular repair is necessary.

### Structural atherosclerosis and functional atherosclerosis

Figure 3 shows a potential vascular mechanism underlying the adaptation to age-related oxidative stress. CIMT is an indicator of structural arterial stiffness while CAVI is an indicator of functional arterial stiffness. Circulating CD34-positive cells are required for progression of structural arterial stiffness (active arterial wall thickening)^23^. Thus, circulating CD34-positive cell count could influence the association between CIMT and CAVI. We found a significant positive association between CIMT and CAVI only among participants with higher circulating CD34-positive cell count (at or above the median) (Fig. 3a/Fig. 3d)^24^. Functional arterial stiffness indicates structural arterial stiffness only among participants with enough CD34-positive cells.

### Functional atherosclerosis and circulating CD34-positive cells

Since CIMT, an indicator of structural arterial stiffness, does not reflect the influence of deficient vascular repair, a parameter that reflects the influence of deficient vascular repair is necessary. Since deficiency of circulating CD34-positive cells is the main cause of deficient vascular repair, such a parameter should be associated with circulating CD34-positive cell count among participants with low circulating positive cell count (below the median). We found a significant inverse association between CAVI and circulating CD34-positive cell count in participants with low circulating CD34-positive cell count (below the median) (Fig. 3b/Fig. 3e)^24^. CAVI is an indicator of functional atherosclerosis that reflects endothelial deficiency, which could not be evaluated with CIMT.

### Endothelial repair activity in relation to circulating CD34-positive cell count stratified by the median value

Aggressive endothelial repair related to hypertension leads to no association between platelet and circulating CD34-positive cell counts^22^. Among participants with high circulating CD34-positive cell count, no significant association between platelet and circulating CD34-positive cell count was observed. Among participants with low circulating CD34-positive cell count, a significant positive association between those two variables was observed (Fig. 3c/Fig. 3f)^24^. Therefore, the investigation of platelet and circulating CD34-positive cell counts also demonstrates that aggressive endothelial repair could be found in participants with high CD34-positive cell count.

This study also showed that circulating CD34-positive cell count stratified by the median value could be an efficient method to classify vascular repair activity^24^. Participants with low circulating positive cell count (below the median) might have lower vascular repair activity than participants with high circulating CD34-positive cell count (at or above the median).

### Structural arterial stiffness and chronic kidney disease (CKD)

Since bone marrow-derived CD34-positive cells play an important role in vascular repair^37^, reduced bone marrow activity should lower the risk of the progression of structural atherosclerosis. Anemia due to reduced production of hemoglobin is a well-known complication of CKD^38^. Reduced bone marrow activity is a well-known complication of CKD, and CKD is reported to be associated with carotid structural atherosclerosis^26^. Since circulating CD34-positive cell count stratified by the median value could be an efficient tool for classifying vascular repair activity^24^, we investigated the association between CIMT and CKD by circulating CD34-positive cell count. CIMT was significantly positively associated with CKD only among participants with high circulating CD34-positive cell count (Fig. 4)^25^. Therefore, circulating CD34-positive cells are mandatory for the development of CIMT, even among participants with CKD, which leads to lower bone marrow activity.

### Association between platelets and hypertension in relation to circulating CD34-positive cell count stratified by the median value

Increased peripheral resistance is a known cause of hypertension. Angiogenesis plays an important role in peripheral resistance via maintenance of the microcirculation. Vascular endothelial growth factor (VEGF) polymorphisms, which play an important role in the progression of angiogenesis, are inversely associated with hypertension^39^.

While CD34-positive cells contribute to the development of structural atherosclerosis, they also contribute to the maintenance of the microcirculation by promoting angiogenesis^12^ and neovascularization^13^. Thus, participants with activated vascular repair who have a shortage of circulating CD34-positive cells might be at risk for hypertension because of the lower capacity to reduce peripheral resistance. Platelet count could act as an indicator of vascular maintenance activity^22^. Platelet count was positively associated with hypertension only among participants with low CD34-positive cell count (below the median)^35^.

This study indicates that CD34-positive cells contribute to the development of structural atherosclerosis^2,4,23,24^. CD34-positive cells might contribute to the prevention of hypertension possibly via maintenance of the microcirculation.

### Gamma-glutamyl transpeptidase (γ-GTP), hypertension, and structural atherosclerosis in relation to circulating CD34-positive cell count stratified by the median value

Aging is a process associated with increasing oxidative stress^40,41^. Since oxidative stress induces hypertension^28^ and atherosclerosis^29^, the magnitude of oxidative stress could play a crucial role in the associations among hypertension, atherosclerosis, and circulating CD34-positive cell count.

Since serum γ-GTP is reported to be a marker of oxidative stress^30,31^, we used γ-GTP to evaluate the influence of oxidative stress on hypertension and atherosclerosis (Fig. 5). No significant association between γ-GTP and hypertension was observed in participants with high CD34-positive cell count (at or above the median), but a significant positive association was observed in participants with low CD34-positive cell count (below the median)^27^. In addition, a significant association between γ-GTP and structural atherosclerosis was observed in participants with high CD34-positive cell count (at or above the median). No significant association was observed in participants with low CD34-positive cell count (below the median)^27^.

CD34-positive cells contribute to both the development of structural atherosclerosis^2,4,23,24^ and maintenance of the microcirculation^12,13^. In participants with sufficient vascular repair capacity, oxidative stress might lead to both atherosclerosis and angiogenesis (including vascularization), which prevent hypertension by reducing peripheral vascular resistance. In individuals without sufficient vascular repair capacity, oxidative stress does not lead to atherosclerosis and angiogenesis (including vascularization), resulting in continued hypertension.

### Potential vascular mechanism underlying age-related physical changes

Figure 3 shows the potential vascular mechanism underlying age-related physical changes. Aging is a process associated with increasing oxidative stress^40,41^. Hypoxia increases oxidative stress^42^. To compensate for a lower oxygen supply, the body might induce hypertension, which increases the supply of oxygen via the existing vascular system. However, oxidative stress itself and hypertension injure the endothelium. With endothelial injury, components of endothelium activate platelets exposed to peripheral blood (Fig. 3-(1))^43,44^. Those activated platelets induce proliferation of CD34-positive cells^2^. Bone marrow-derived CD34-positive cells play a crucial role in vascular repair^37^. However, aging leads to lower bone marrow activity^45,46^. If bone marrow could be activated efficiently, a sufficient number of circulating CD34-positive cells could be supplied (Fig. 3-(2)). Since CD34-positive cells contribute to both the development of structural atherosclerosis^2,4,23,24^ and maintenance of the microcirculation by promoting angiogenesis^12^ and neovascularization^13^, peripheral vascular resistance could be reduced. Therefore, hypertension could disappear among older participants with a sufficient number of circulating CD34-positive cells. However, if the influence of decreased bone marrow activity due to aging is strong, a shortage of circulating CD34-positive cells could occur (Fig. 3-(3)). Due to a shortage of CD34-positive cells, structural atherosclerosis does not develop, but the microcirculation might become disrupted, which elevates oxidative stress and might lead to residual hypertension.

### Summary of the potential biological reactions to hypoxia and oxidative stress

**Figure 7:**
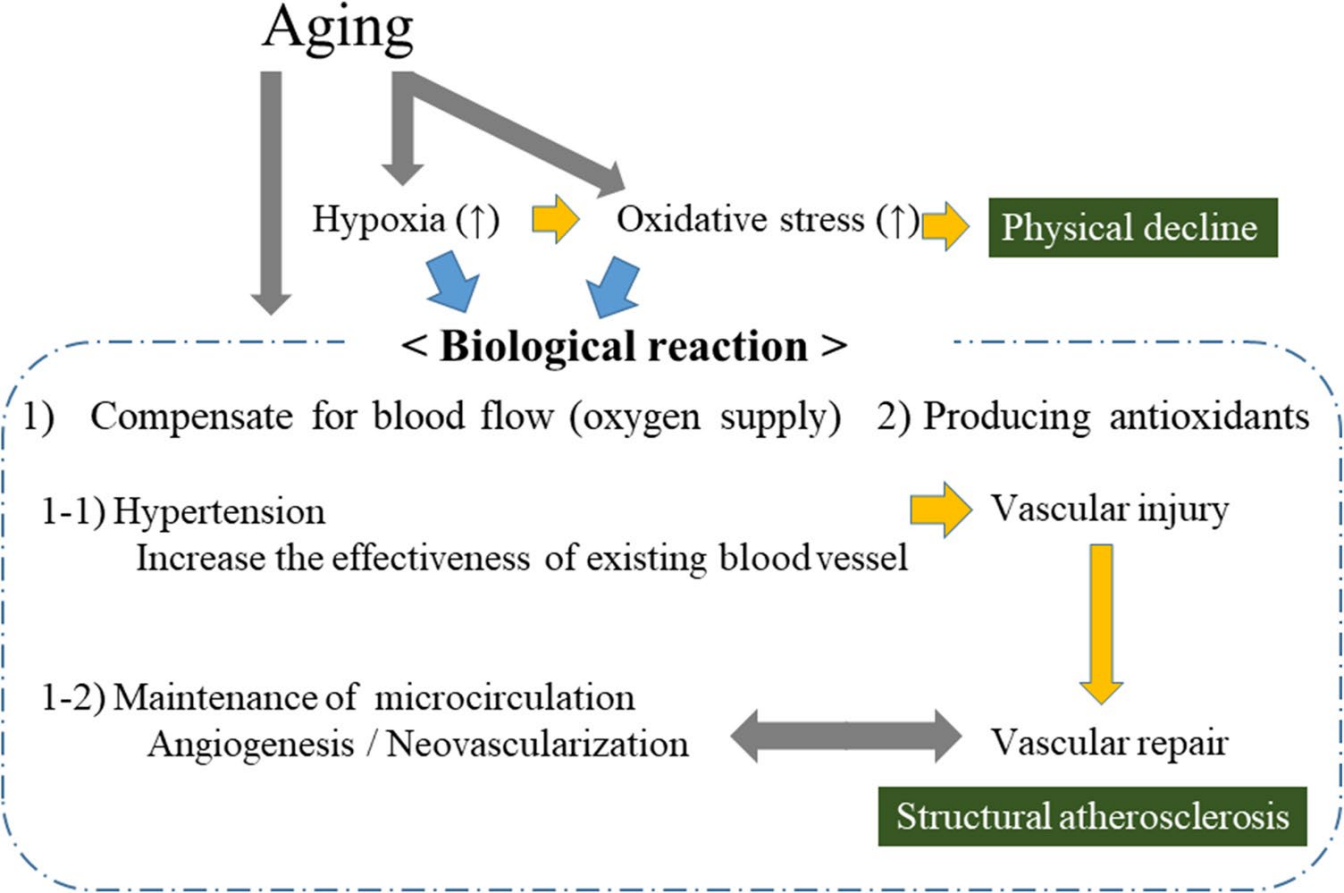
Potential biological reaction for hypoxia and oxidative stress.

Figure 7 shows a summary of the potential biological adjustments to hypoxia and oxidative stress. Along with the process of aging, hypoxia and oxidative stress increase^40–42^. The biological reactions to hypoxia and oxidative stress aim to adjust for age-related physical changes. The first reaction involves mechanisms intended to compensate for decreased blood flow (oxygen supply) (Fig. 7-(1)). i.e., hypertension and maintenance of the microcirculation via angiogenesis and neovascularization. By increasing the effectiveness of existing vessels, hypertension increases the oxygen supply (Fig. 7-(1)-(1-1)). By increasing the vascular network (maintenance of the microcirculation), angiogenesis and neovascularization could also increase the oxygen supply (Fig. 7-(1)-(1-2)). However, hypertension injures the vasculature, which increases the need for vascular repair. Aggressive vascular repair leads to structural atherosclerosis. CD34-positive cells contribute to both maintenance of the microcirculation and development of structural atherosclerosis. Since aging is a process that decreases the production of CD34-positive cells^45,46^, development of structural atherosclerosis partly indicates residual capacity to maintain the microcirculation. Therefore, hypertension and development of structural atherosclerosis might act as indicators of adaptation to age-related physical changes. Antioxidant production by reticulocytes^47^, for example, also plays an important role in adaptation to age-related physical changes (Fig. 7-(2)).

### Handgrip strength and hypertension in relation to circulating CD34-positive cell count

Aging is a process associated with increasing oxidative stress^40,41^. Oxidative stress reduces muscle strength^48^. Hypertension is induced to compensate for a reduced oxygen supply with the existing vasculature. Hypertension is reported to be positively associated with handgrip strength^33,34^. However, hypertension is also associated with an abnormal microcirculation^49^. Therefore, the status of the microcirculation is also an important factor related to age-related muscle strength reduction^50,51^. Since CD34-positive cells contribute to the maintenance of the microcirculation^12,13^ and have a beneficial effect on oxygen supply, the beneficial influence of hypertension on maintaining muscle strength should be limited to participants with a sufficient number of circulating CD34-positive cells. This is the reason why a positive association between handgrip strength and hypertension was only observed in participants with high circulating CD34-positive cell count (at or above the median) (Fig. 6)^32^.

Handgrip strength is reported to be inversely associated with fatal cardiovascular disease^52^. Hypertension is the strongest cardiovascular risk factor^15^ and is reported to be positively associated with handgrip strength^33,34^. Those findings seem paradoxical. However, our previous study revealed that the positive association between handgrip strength and hypertension was limited to participants with high circulating CD34-positive cell count^32^, which could explain this paradoxical phenomenon. CD34-positive cells play an important role in vascular repair^1–6^ and maintenance of the microcirculation^12,13^. Circulating CD34-positive cell count is reported to be inversely associated with cardiovascular disease^7–9^. Therefore, the beneficial effect of maintaining muscle strength that hypertension possesses should be supported by active endothelial repair.

### Perspective

Generally, hypertension and structural atherosclerosis are regarded as health disturbances. However, both of those factors are biological reactions against hypoxia and increased oxidative stress. Biological reactions should have beneficial effects. In other words, a deficiency in hypertension and structural atherosclerosis that leads to coordinated maintenance of the vasculature could be disadvantageous for health. The present study indicates that circulating CD34-positive cells might coordinate the beneficial influence of hypertension and structural atherosclerosis on health. Since bone marrow-derived circulating CD34-positive cells play an important role in vascular repair^37^ and aging is a process that decreases CD34-positive cell production^45,46^, CD34-positive cell production could be more important in an aged society.

### Limitation

Only a few studies have been conducted to assess this concept. Circulating CD34-positive cells could influence the association between triglycerides and hypertension^53,54^. Circulating CD34-positive cells also could influence the association between high-density lipoprotein cholesterol and hypertension^55^. Furthermore, adult height could be associated with CD34-positive cell production^56,57^. In addition, thyroid hormone has been reported to regulate bone marrow-derived hematopoietic stem cells^58^. The absence of thyroid cysts could act as a marker of latent thyroid damage^59^, which could influence thyroid function. The presence of thyroid cysts influences the association between structural atherosclerosis and hypertension^60^. Therefore, those factors also could influence the coordination of vascular maintenance. More detailed investigations based on this concept are strongly required. Due to financial and technical reasons, we limited the measurement of CD34-positive cell count to men aged 60–69 years. Further investigation with larger sample could be informative.

## Conclusion

This study about circulating CD34-positive cells describes a novel mechanism that explains the beneficial effect of hypertension and the development of structural atherosclerosis. Both of those factors are biological reactions against hypoxia and increased oxidative stress. Aggressive endothelial repair increases both functional and structural atherosclerosis, while deficient endothelial repair relates to furthers functional atherosclerosis but not structural atherosclerosis. Circulating CD34-positive cells, which play an important role in endothelial repair, coordinate the beneficial effect of hypertension and structural atherosclerosis on vascular health.

## Data Availability

We cannot publicly provide individual data due to participant privacy, according to ethical guidelines in Japan. Additionally, the informed consent obtained does not include a provision for publicity sharing data. Qualifying researchers may apply to access a minimal dataset by contacting the office of data management at ritouken@vc.fctv-net.jp. Information for where data request is also available at https://www.genken.nagasaki-u.ac.jp/dscr/message/ and http://www.med.nagasaki-u.ac.jp/cm/.
